# Epidemiological risk factors and the geographical distribution of eight *Mycobacterium* species

**DOI:** 10.1186/s12879-021-05925-y

**Published:** 2021-03-12

**Authors:** Maura J. Donohue

**Affiliations:** United States Environmental Protection Agency, Office of Research and Development, Center of Environmental Solutions and Emergency Response (CESER), 26 W. Martin Luther King Dr. Mail Stop 587, Cincinnati, OH 45268 USA

**Keywords:** Nontuberculous mycobacteria (NTM), Epidemiology, Patient prevalence, United States, *Mycobacteriumabscessus*, *M. avium* complex (MAC), *M. chelonae*, *M. fortuitum*, *M. gordonae*, *M. kansasii*, *M. mucogenicum*, *M. peregrinum*

## Abstract

**Background:**

Nontuberculous mycobacteria (NTM) are environmental bacterium that may cause and/or compound respiratory diseases in humans. There are over a hundred NTM species with varying pathogenicity’s Therefore, it is necessary to characterize the populations at risk for each species.

**Methods:**

Demographic (age, sex, and state of residence) and microbiological data from 2014 were extracted from Mississippi, Missouri, and Ohio disease surveillance systems. NTM species with > 50 reports were included in the analysis. Patient sex, age, and incidence rates were generated for each of the following NTM species: *M. abscessus*, *M. avium* complex (MAC), *M. chelonae*, *M. fortuitum*, *M. gordonae*, *M. kansasii*, *M. mucogenicum*, and *M. peregrinum*.

**Results:**

Analysis by sex showed that *M. chelonae*,*M. fortuitum*, *M. gordonae,*and *M. kansasii* had significantly higher rates in males than females. Age was not associated with patient rates for several specific NTM species e.g., *M. chelonae*. Mississippi had the highest patient’ rates for *M. avium*, *M. gordonae*, *M. kansasii*, and *M. chelonae*. Ohio had the highest patient’ rates for *M. abscessus*, *M. mucogenicum*, and *M. peregrinum*. The highest patient’s rate for *M. fortuitum* was observed in Missouri.

**Conclusion:**

This study showed that NTM infection occurred more frequently in males. The highest rates were observed in Mississippi for most of the NTMs studied. Age was not a strong risk factor for some of the NTM species.

**Supplementary Information:**

The online version contains supplementary material available at 10.1186/s12879-021-05925-y.

## Background

Nontuberculous mycobacteria (NTM) are ubiquitous environmental bacteria present in soils and water. Therefore, human exposure to these bacteria can occur through daily activities such as showering, bathing, and gardening. Nontuberculous mycobacteria are opportunistic pathogens that can cause a wide range of non-communicable skin and soft tissue infections as well as respiratory diseases. The high-risk population for NTM infections includes individuals with Cystic Fibrosis (CF) [1], bronchiectasis, emphysema, chronic obstructive pulmonary disease (COPD) [2], and weakened immune system. Among the various manifestations of NTM-linked infections, pulmonary NTM disease is the severest form because it lacks a cure [3].

In the U.S., over the past two decades, numerous reports demonstrate that prevalence rates for NTM infections are increasing [4–7]. Despite NTM infections being unfamiliar to the at-large public, possible factors contributing to the apparent increase are the aging U.S. population, better diagnostics, and improved reporting to disease registries. Additionally, environmental and or host factors may have shifted, introducing these opportunist pathogens to a newly susceptible population.

Physiological risk factors linked to an increased risk for infection include age [8] and, among women, rheumatoid arthritis [4, 9], thoracic skeletal abnormalities [10] and steroid usage [11]. There are few papers that identified specific exposure risk factors. However, soil contact in the US southern states [11], swimming pool usage among CF patients [12] and aerosolized shower water [13] have a demonstrated a potential association with pulmonary NTM disease. The same is true for environmental risk factors such as evapotranspiration, climate, and the impact of soil minerals, such as copper and manganese [14]. Of those studies that have tried to identify environmental risk factors, there is a lack of agreement as to the major contributor.

Diagnosis of pulmonary NTM disease involves a multifaceted approach that considers a hierarchy of evidence [3] (e.g., sign/symptoms, microbiological cultures, and chest x-rays). Most environmental risk factor studies focus on the disease state giving little consideration to the specific species causing the disease. Lumping all NTM species together may confound the results with minimal progress made in identifying other risk factors.

An integrative demographic analysis of data from case reports may help identify the exposure sources and risk factors (e.g., age, sex, NTM species) that contribute to the United States’ infection/disease rate. However, this type of demographic segmentation or multivariant analysis is infrequent. It requires a large amount of data to achieve statistical significance between the variables. Unfortunately, the U.S. lacks a good data source to support such an analysis on a national scale. The few datasets that do exist are confounded by ambiguous species identification and incomplete patient demographic information. However, some state information does exist, that would allow a targeted analysis.

The goal of this study was to identify human susceptibility patterns associated with demographic data sex, age, and location for eight commonly isolated NTM species, *M. abscessus*, *M. avium* complex (MAC), *M. chelonae*, *M. fortuitum*, *M. gordonae*, *M. kansasii*, *M. mucogenicum*, and *M. peregrinum.* Identifying the demographics associated with NTM infections will enable targeted mitigation strategies to be enacted to protect the most impacted part of the population.

## Methods

### Data collection strategy

Nontuberculous mycobacteria infection/diseases are not a Nationally recognized infection/disease reportable to the United States Center of Disease Control and Prevention’s (CDC) National Notifiable Disease Surveillance System (NNDSS). Therefore, very few states collect surveillance data on Mycobacterial disease. Mississippi, Missouri, and Ohio are three states that do. These three states are in different geographical locations: East North Central (Ohio), West North Central (Missouri), and East South Central (Mississippi).

The State health departments compiled the number of patient-NTM infections by species, patient sex (male and female), and patient age category (< 1–39, ≥40–49, ≥50–59, ≥60–69, ≥70–79, ≥80, & unknown) for the 2014 calendar year. The Donohue et al. 2018 paper contains information on each state’s data sources and NTM identification methods [15]. The data provided to EPA contained no patient personal identifiers. In this report, the term “case” refers to a unique individual whose specimen was culture-positive for a specific NTM species. Additionally, the American Thoracic Society/Infectious Disease Society of America (ATS/IDSA) NTM microbiological or disease criteria were not applied to the patient cases. This was done so that comparisons to previous studies that use could be performed. This study was exempt from the Institution Review Board review by U.S. EPA.

The provided data were used to evaluate infection patterns associated with a patient based on sex, age, and state of residence. Only cases that categorized the individual species of NTM were used; no NTM group or complex-level were included other than those ascribed to the *Mycobacterium avium* Complex (MAC). Additionally, a minimum of 50 cases need to be assigned to a specific species to obtain enough statistical strength. Of the 30-plus species, groups, and complex designations provided by the health departments, only eight NTM species met the study criteria. These eight species were *M. abscessus,* MAC, *M. chelonae, M. fortuitum*, *M. gordonae*, *M. kansasii, M. mucogenicum*, and *M. peregrinum.*

### Analysis

The U.S. 2014 Census demographic data (total population, age ranges, and segmentation by sex for the population) were collected from the U.S. Census Bureau for each state [16]. Initially, case numbers for each species/complex were converted to patient prevalence rates (cases per 100,000 persons) for each sex (male and female). Next, each sex infection rate was segmented by age category (< 1–39, ≥40–49, ≥50–59, ≥60–69, ≥70–79, ≥80, & unknown). Next, crude rates were first adjusted by sex (male =0.49 and female 0.51), then by age. The standardized age-adjusted prevalence rates were applied to the U.S. 2000 population-weighted age distribution [17]. Lastly, the sex-age adjusted rates were segmented by the state of residence (Mississippi, Missouri, and Ohio).

As described above, case numbers were converted to crude patient-prevalence rates. The crude prevalence was calculated as the number of cases divided by the state’s population using July 1, 2014 population estimates. The age-specific rates were calculated using the following equation: (total number of cases in each age group ÷ state’s 2010 population for the same age group). The age-adjusted prevalence rates were standardized against the U.S. 2010 age distribution [17].

### Data source

The Mississippi, Missouri, and Ohio Health Departments provided the U.S. EPA with the number of NTM specimen for the year 2014. Mississippi data are publicly available at their website: http://msdh.ms.gov/msdhsite/_static/14,0,261.html. Raw data is in supplementary file 1.

### Statistical analyses

The percent change values, and regression analysis of the age-adjusted prevalence rates were calculated using Microsoft Excel (version 365 ProPlus). SigmaPlot version 14.0 (Systat Software San Jose, CA) was used for the Chi-square and Mann-Whitney U-test to determine statistical significance. An alpha of 0.05 was used.

## Results

In 2014, 2787 NTM cases were detected in the three states. Of these, 2405 had information on infective species, age, and sex of the infected individual. The NTM species/complex most frequently isolated from human specimens were MAC (*n* = 1312), *M. gordonae* (*n* = 498), *M. fortuitum* (*n* = 188), *M. chelonae* (*n* = 100), *M. mucogenicum* (*n* = 87), *M. kansasii* (*n* = 85), *M. abscessus* (*n* = 77) and *M. peregrinum* (*n* = 58). These eight NTM species/complex represented 93% of the total cases reported to the state health departments. The eight NTMs were composed of three slow-growing groups (MAC, *M. gordonae*, and *M. kansasii*) and five rapid-growing species (*M. fortuitum*, *M. chelonae*, *M. abscessus*, *M. mucogenicum*, and *M. peregrinum*). Not all states had cases with *M. peregrinum* e.g., Mississippi*.*

### Species infection rates by NTM species and sex

**Fig. 1:**
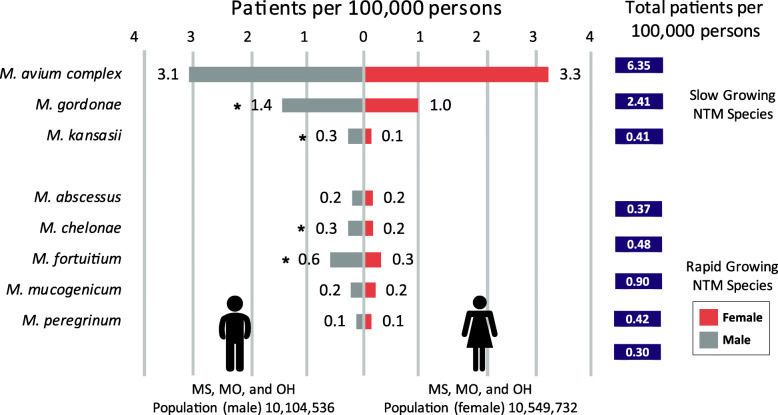
Infection Rates by Species and Sex. * Statistically Significant χ^2^

The number of cases from all three states for males was 1286, a crude rate of 12.7 per 100,000 persons for all NTM species combined. For females, 1119 cases or a crude rate of 10.6 per 100,000 persons was identified for all three states combined. Comparison of the sexes by NTM species demonstrates differences in infection rates are observed for some of the species (Fig. 1). For the slow-growing species, *M. gordonae* and *M. kansasii*, significantly higher infection rates were observed among males as compared to females *P* = < 0.001. As for MAC, the species with the largest number of cases, the infection rate was similar for males (3.1 patients per 100,000 persons) and females (3.3 patients per 100,000 persons).

For the five rapid-growers, three species, *M. abscessus*, *M. mucogenicum*, and *M. peregrinum*, had similar infection rates for males and females. However, *M. fortuitum* and *M. chelonae* had significantly higher infection rates in males than females, *P* = < 0.001 and *P* = 0.034, respectively. The infection rates for the *M. avium* complex and *M. gordonae*, slow-growing species, were the highest among the species evaluated (Fig. 1).

### Infection rates by age

**Fig. 2:**
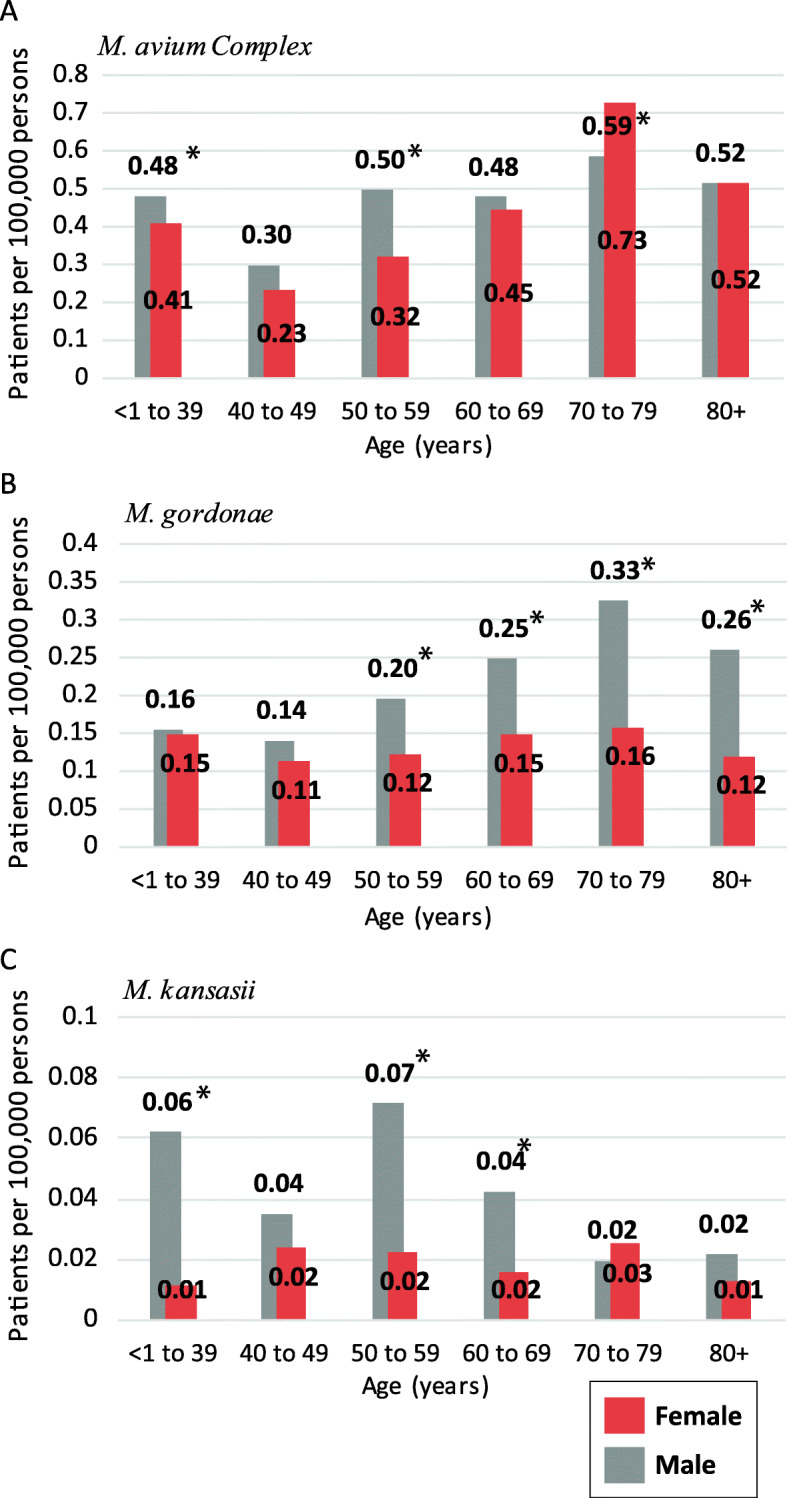
Slow-growing species age-specific rates by gender **a** *M. avium* Complex (MAC), **b** *M. gordonae*, and **c** *M. kansasii*. * indicate statistical significance between the sexes within a specific age group

As demonstrated, infection rates differ between the sexes, depending on the species. The age category may also influence the infection rate. By maintaining the sex segmentation in combination with age, the analysis can reveal if there are age-specific differences between the sexes. The slow-growing species had a unique sex-age profile. (Fig. 2). The MAC infection rates increased with age for both males and females (Fig. 2a). For males, the infection rates were statistically higher than those for females within the following age categories, < 1 to 39, 50 to 59, and 80+ age *P* = 0.049, < 0.001, and < 0.001. The sex difference was statistically significant for females only for those in the 70–79 years age group, *P* = < 0.001.

The *M. gordonae* sex-age distribution (Fig. 2b) showed substantial infection rate differences by sex. The *M. gordonae* male infection rates are higher than the female rates, in the 50 to 59 age group, and the sex differences continued to increase as age increased. The female infection rate for *M. gordonae* was not observed to increase with age. *M. kansasii* was detected in far fewer cases per 100,000 persons than the other two species (Fig. 2c), indicating a lower infection rate. In general, the male rates exceeded those for females. The difference was significant for the 50–69 age category, *P* = < 0.001). The R^2^ value in females, *R*^*2*^ = 0.0003, is lower compared to the males *R*^*2*^ = 0.4922. Inferring infection rate changes is not influenced significantly by the age of the patient.

**Fig. 3:**
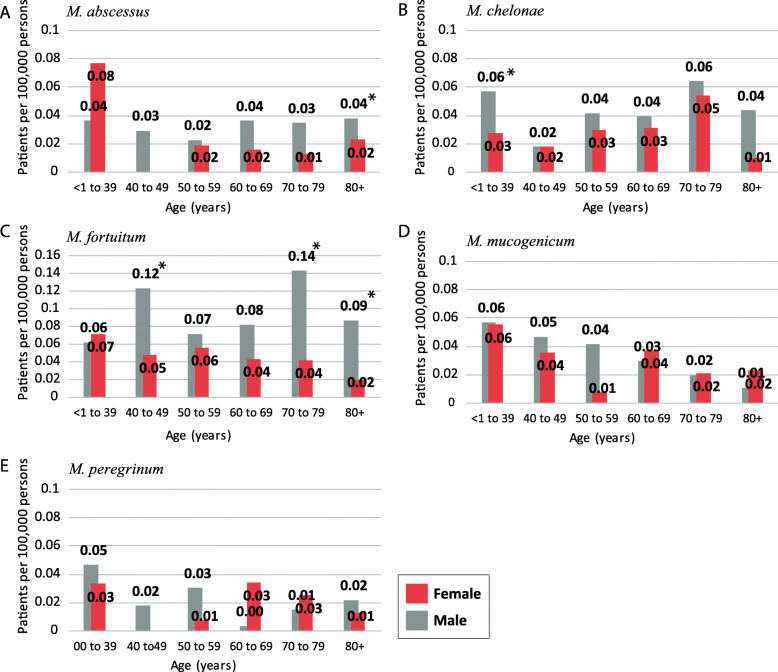
Rapid-growing species age-specific rates by sex **a** *M. abscessus*, **b** *M. chelonae*, **c** *M. fortuitum*, **d** *M. mucogenicum*, and **e** *M. peregrinum*. * indicate statistical significance between the sexes within a specific age group

The five rapid-growing species had far fewer cases per 100,000 persons for all three states than the slow-growing species. In general, the species-specific rates were not affected by age or sex of the patient (Fig. 3). The female patient rate for *M. abscessus* did not vary much by age, except for the < 1–39 year age category. In males, the *M. abscessus* infection rate did not alter by age (Fig. 3a). The *M. chelonae* sex-age distributions are similar between the sexes. The exception, for male < 1–39 year old rate was significantly higher than the females < 1–39 year old category. The *M. fortuitum* analysis (Fig. 3c) resulted in an interesting distribution where the male infection rate was significantly higher, *P* = < 0.001–0.007, than that for females for several age categories.

Table 1 ranks species-specific rates from most to least associated to age, as determined by linear regression analysis. Many species related infections rates decreased with age for males: *M. mucogenicum*, *M. kansasii*, and *M. peregrinum* and for females: *M. fortuitum, M. mucogenicum*, and *M. abscessus*. Other species-specific infection rates increased with age: *M. avium* (male and female), and *M. gordonae* (male). Lastly, there were several species that had no change, or no trend associated with age when separated by sex; these species had an *R*^*2*^ = < 0.2.

**Table 1:** Ranking of species-specific age-adjusted rates by sex

Rank	Male	***R***^**2**^	Trend	Female	***R***^**2**^	Trend
1	*M. mucogenicum*	0.9942	Descending with age	*M. fortuitum*	0.8160	Descending with Age
2	*M. gordonae*	0.7439	Ascending with age	*M. avium*	0.4524	Ascending with Age
3	*M. kansasii*	0.4922	Descending with age	*M. mucogenicum*	0.3289	Descending with Age
4	*M. peregrinum*	0.3338	Descending with age	*M. abscessus*	0.2222	Descending with Age
5	*M. avium*	0.3313	Ascending with age	*M. chelonae*	0.0055	No Trend
6	*M. abscessus*	0.1206	No trend	*M. kansasii*	0.0033	No trend
7	*M. fortuitum*	0.101	No trend	*M. gordonae*	0.0026	No trend
8	*M. chelonae*	0.0533	No trend	*M. peregrinum*	0.0000	No trend

### Infection rates by the state of residence

At a state-level (Table 2), Mississippi experienced the highest age-adjusted patient-rate per 100,000 persons for MAC, *M. kansasii*, *M. chelonae*, and *M. fortuitum*. In comparison, Missouri had a higher rate for the *M. fortuitum specie*. Ohio experienced that highest rates for *M. abscessus*, *M. mucogenicum*, and *M. peregrinum*. In Mississippi, *M. abscessus* cases were rarely reported, and there were no reports associated with *M. peregrinum*. Ohio had the most cases for *M. peregrinum* (*n* = 52).

**Table 2:**
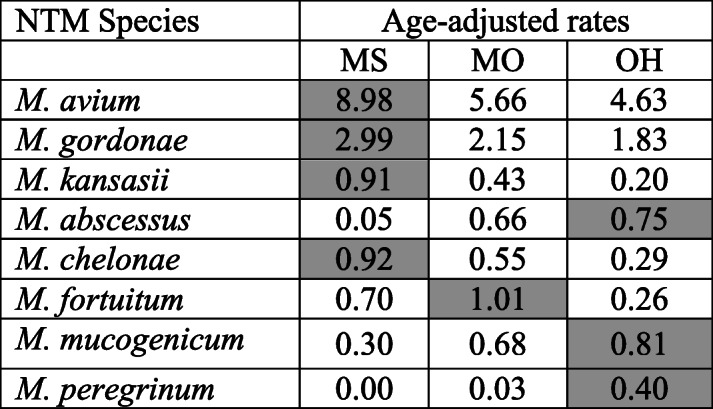
Age-adjusted rates by State

The cell with the highest species-specific rate is highlighted

**Fig. 4:**
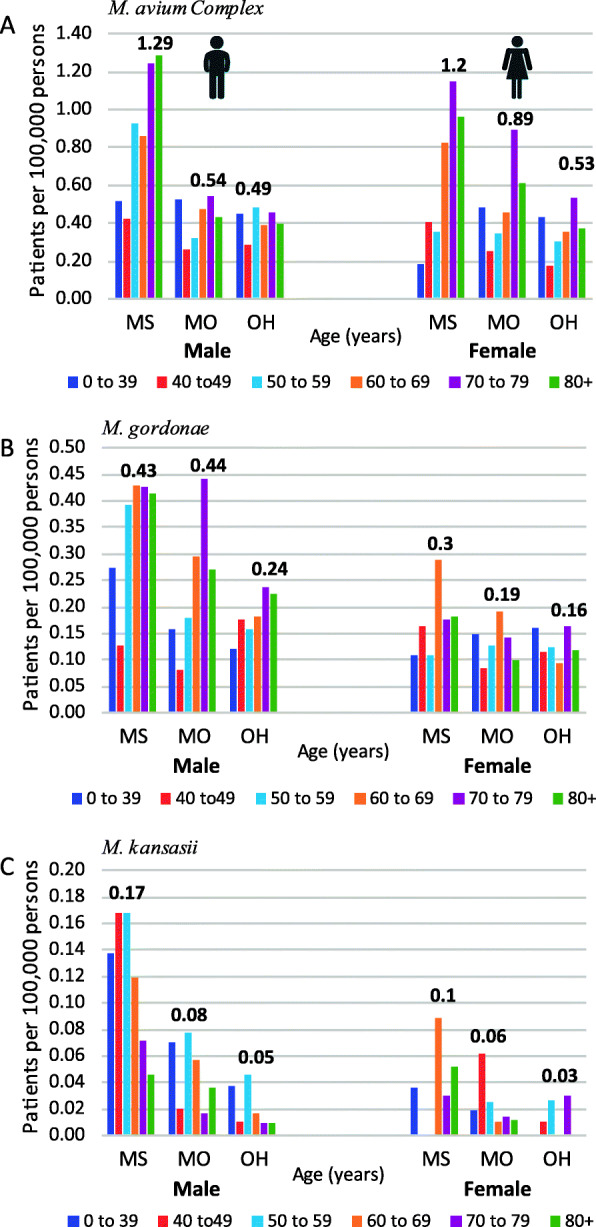
Slow-growing species rate by State, sex, and age **a** *M. avium* Complex (MAC), **b** *M. gordonae*, and **c** *M. kansasii*. The highest rate for each sex by state is labeled

The state of residence appeared to influence the distribution of infection rates while maintaining the separation of sex and age categories for many of the reported NTM species. Male infection rates of MAC have different patterns among the states. In comparison, female MAC infection rates by the state have similar patterning to each other (Fig. 4a). The *M. gordonae* age-specific rate pattering does vary by state for males. While female rates by state are unsystematic in profile (Fig. 4b). On the other hand, the sex-age distribution varied considerably across the states for *M. kansasii*. Higher rates for the 50 to 69-year-old males were consistent across the three states (Fig. 4c).

**Fig. 5:**
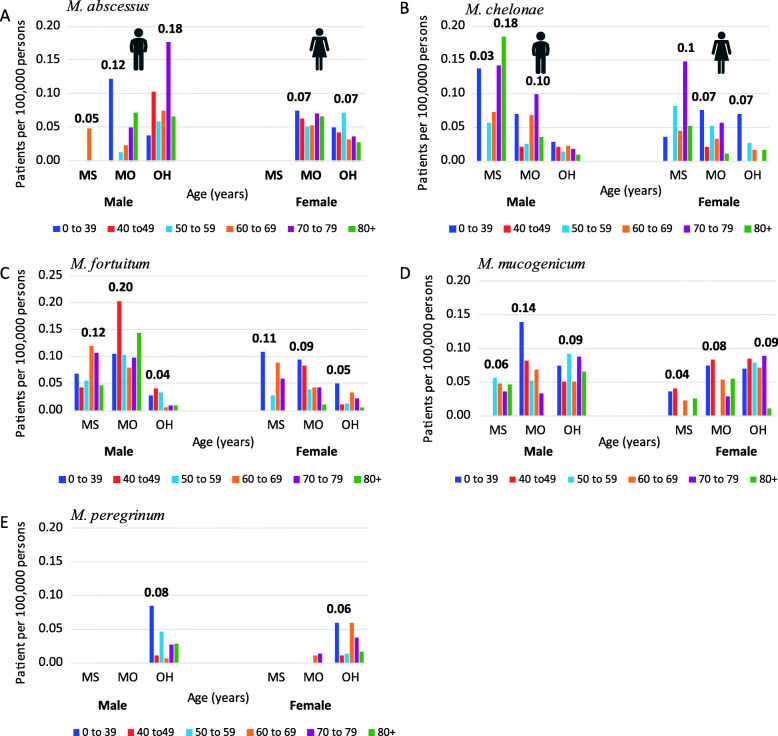
Rapid-growing species rates by state, sex, and age. **a** *M. abscessus*, **b** *M. chelonae*, **c** *M. fortuitum*, **d** *M. mucogenicum*, and **e** *M. peregrinum*. The highest rate for each sex by state is labeled

The rapid-growing species are not as universally isolated from humans in these states as the slow-growing MAC and *M. gordonae* (Fig. 5). Figure 5 shows how regional differences influence the distribution of infection rates by species and state, again, maintaining the separation of sexes and age categories. The age most affected by a specific species is highly variable among states and sexes. This suggests that these species are more opportunistic (requiring specific host factors for infection) than the slow-growing species. These results also suggest that risk factors other than age are involved.

## Discussion

Describing the at-risk populations for a disease is a long-standing practice that helps identify vulnerability patterns. The identified risk factors can assist practitioners with diagnosis, selecting the treatment approach for the disease, and providing guidance for at-risk populations. Seven of the eight NTM species studied can cause infections or diseases in humans [3]. However, the pathogenicity and the specimen isolation frequency are not equal across species. Of the eight NTM species, only the species *M. gordonae* is considered non-pathogenic to either humans or animals.

Knowing the sex with the highest infection rate can potentially reveal underlying physiological (10), genetic, behavioral (12), and or occupational factors [18, 19] that increase the risk for disease. In the 1980s and 1990s, NTM related illnesses were associated more with males who represented 57 to 63% of the population-normalized case reports for the U.S. [20, 21]. In the early 2000s, Winthrop et al. first reported a shift in the caseload based on sex, with females taking over the lead in Oregon [22]. Other researchers have observed this same trend (6). This demographic shift based on sex is intriguing because it reveals that a fundamental change in vulnerability may have occurred (e.g., a new exposure route, new morbidity factor, or some other unknown factor).

Clinically, pulmonary NTM diseases manifest themselves differently for females versus males. Females develop manifestations known as Lady Windermere disease (mid-lobe infections), while males experience the fibrocavitary NTM form of the disease (upper-lobe infections) [23]. These varying clinical displays support the possibility that either physiological and or genetic variables are responsible for shaping disease expression within an individual.

In this study, total population-normalized cases by sex were nearly equivalent and had similar values as those reported by Smith et al. [24]. Differences by sex are expected at the species-level. Male and female MAC patients had comparable infection rates, indicating that exposure routes and morbidity factors may be similar. Similar male and female MAC patient rates are different from previously reported outcomes in O’Brien et al. and Butler et al. Table 3 [20, 21]. Males also had statistically significantly higher infection rates than those for *M. gordonae*, *M. kansasii*, *M. chelonae*, and *M. fortuitum* (*P* = < 0.001, 0.034, < 0.001, and < 0.001), respectively.

**Table 3:** Reported adjusted rates by species and sex

	1980’s		1990’s		2000’s	
	O’Brien et al. 1982^a^		Butler and Crawford 1999		This Study	
Adjusted-Reports per 100,000 persons	Male	Female	Male	Female	Male	Female
*M. avium* complex (MAC)	0.31	0.29	1.98	1.16	3.08	3.27
*M. gordonae*			1.06	0.63	1.43	0.98
*M. kansasii*	0.1	0.04	0.17	0.08	0.28	0.13
*M. abscessus*			0.01	0.01	0.21	0.16
*M. chelonae*	0.02	0.01	0.08	0.07	0.28	0.20
*M. fortuitum*	0.06	0.05	0.32	0.19	0.59	0.31
*M. mucogenicum*			0.001	0.001	0.22	0.20
*M. peregrinum*			0	0	0.14	0.16
Population (thousands)	110,053.1^b^	116,492.6^b^	121,239.3^c^	127,470.5^c^	10,104,536	10,549,732

^a^ Appendix A- report numbers; ^b^1980 U.S. Census; ^c^1990 U.S. Census

Interestingly, male dominance for these species had been identified earlier, but was not a focus in past demographic studies (Table 1) [20, 21]. The sex-based bias suggests that males may be participating in some activities or occupations that differ from those for females or have different associated morbidity factors that predispose them to infection. Work by Ford et al. showed that each NTM species had an association with some unique co-morbidity factors [25]. For example, chronic lung disease and bronchiectasis are common among patients with *M. abscessus* complex infections, while COPD is common among patients with *M. kansasii* infections. These findings demonstrate that specific morbidity factors may influence the populations susceptible to infections from specific NTM species in addition to sex and age.

Adding age to the species-sex observations further refines the “at risk” population analysis. Although a strong age association was observed for some species, not all infection rates were impacted by age. This development is interesting because it demonstrates that there may be other factors that influence NTM infection risk, e.g., *M. kansasii*.

This study is the first to report the disparate sex-age pattern associated with *M. kansasii*, where the male case distribution differs significantly from the female age-related distribution (Fig. 4c). O’Brien et al. report disagrees with the results in this current study. However, this disagreement is due to differences in the age groupings [21]. Ahn et al. paper reported a similar age distribution for males, but the female age rate pattern observed in this study was different (Fig. 4c) [26]. Another pattern observed in this study was the male-age dominance within the *M. fortuitum* and *M. gordonae* data. In both cases, the male infection rate was significantly higher than the female rates. This disproportional sex-age distribution could be due to occupational or underlying behavioral factors that have increased male susceptibility. Clinically, both *M. fortuitum* and *M. gordonae *species are not strongly pathogenic, and treatment is rarely required. Another interesting aspect for *M. fortuitum* and *M. gordonae* is that, unlike *M. mucogenicum*, they lack a high occurrence in potable water [27]. Nevertheless, they are the second and fourth most common species isolated from human specimens [15]. Further analysis involving occupation and or behavioral measures is needed to understand whether additional risk factors are associated with the sex-age related differences.

The environmental occurrence patterns for NTM species across the U.S. are not equal. Due to the wide range of climates in the U.S., it is not surprising that each species might have a unique environmental niche related to state and local conditions. Past epidemiological studies have shown that MAC infections occur more often in the South Atlantic region, whereas *M. kansasii* rates are higher in the west, south-central region [20, 28]. This study involved three states, located in three regions, East North Central (Ohio), West North Central (Missouri), and East South Central (Mississippi). Despite the limited regional representation, geographical differences were detected.

Mississippi, a southern state, that has a hot and humid climate, had the highest MAC rate for both males and females among the three states, and the Missouri female MAC infection rate was higher than that of the male rate (Fig. 4a & b). These changes in MAC distribution by state could indicate that more than one exposure route exists for these species at these geographic locations. The observation of females having a higher MAC rate than males is in agreement with results from other regionally based NTM studies such as Winthrop et al. and Smith et al. that also reported higher female MAC rates than males [22, 24].

In Ohio, a state considered to have a cold to very cold climate, had the highest patient rates for many of the rapid growing species, e.g. *M. abscessus*, *M. mucogenicum* and *M. peregrinum* (Table 2). However, these species’ sex-age by state distribution (Fig. 5a, d and e) were not as stable as the *M. chelonae* and *M. fortuitum* distributions (Figs. 4b and 5c). This is due to the lower number of patient cases (< 100 cases). To obtain a firmer distribution for these species (Fig. 5: *M. abscessus*, *M. mucogenicum* and *M. peregrinum*), a multi-year analysis is necessary to confirm this study’s findings.

The data used in this study came from laboratory reports submitted to the State’s disease surveillance systems. The advantages and limitations of this type of data have been discussed previously [5, 15]. Moreover, ATS/IDSA’s current NTM disease criteria were not applied to the dataset for comparative purposes to past studies. Regardless of a clinician’s stance on disease progression or severity necessary before initiating treatment, the laboratory reports reflect symptoms of high enough concern that the patient visited a doctor, and the doctor ordered the collection of a specimen for analysis. The NTM species causing the infections and the persons they affected are essential details to know when describing the bacteria’s pathogenesis. Therefore, speciation to the species-level is important. It reveals trends that would have been obscured in studies that combine all NTM reports rather than cataloging the cases by species. A recent example regarding the necessity of refining the “at risk” population by species is presented in the Tzou et al. 2020 [13] paper. Shower aerosols exposures would have been missed if the researchers had not age- or fully-adjusted the rates by age, race, and education level [13]. Therefore studies, such as this one are needed to provide refined resolution to the “at risk” population. Population refinement may help both host factor and environmental risk studies to reduce the potential of diminishing factors affecting risk. This “at risk” refinement will also add specificity to diagnostic criteria and better describe each species’ critical risk factors.

## Conclusions

Each NTM species had a unique human susceptibility pattern as associated with sex, age, and geographical location. It is interesting to notice that geography exhibited a strong influence on the sex and age distribution differences associated with the NTMs cases reported. The patient cases observed for *M. avium* were similar for older males and females in Mississippi. However, Missouri’s and Ohio’s *M. avium* cases were more frequent in older females than males. These patient disease rate differences are important because they could indicate more than one environmental exposure route for NTM transmission or an inherent susceptibility to the disease within the state’s population. This study includes states with diverse climates and populations. Based on the observed differences in the susceptible at-risk population, future studies could consider states located on the East and West coast, given that the states in this study were located in the central part of the United States. Additionally, collecting data on how patient disease rates are impacted by pre-existing medical conditions and socioeconomic status would be of value. Such a study could help clarify the populations most at risk for infection and the symptoms that precipitate the need for medical care, thereby identifying confounding variables associated with the host and environmental risk factors.

## Supplementary Information

**Additional file 1.**

## Data Availability

Raw data is in supplementary file 1 and is accessible at data.gov. search: author’s name .

## Abbreviations

ATS: American Thoracic Society
CDC: Center of Disease Control and Prevention
COPD: Chronic Obstructive Pulmonary Disease
CF: Cystic Fibrosis
IDSA: Infectious Disease Society of America
MAC: *Mycobacterium avium* Complex
MI: Mississippi
MO: Missouri
NNDSS: National Notifiable Disease Surveillance System
NTM: Nontuberculous mycobacteria
OH: Ohio
US: United States
